# 肺癌相关单核苷酸多态性的研究进展

**DOI:** 10.3779/j.issn.1009-3419.2011.02.10

**Published:** 2011-02-20

**Affiliations:** 1 250117 济南，山东省肿瘤医院 Shandong Tumor Hospital, Jinan 250117, China; 2 300052 天津，天津医科大学总医院，天津市肺癌研究所，天津市肺癌转移与肿瘤微环境重点实验室 Tianjin Key Laboratory of Lung Cancer Metastasis and Tumor Microenviroment, Tianjin Lung Cancer Institute, Tianjin Medical University General Hospital, Tianjin 300052, China

肺癌是目前全球范围内对人类健康威胁最大的恶性肿瘤之一，关于肺癌的病因学、预防、诊断、治疗等研究已经成为世界各国的重要课题^[1-3]^。已有研究表明遗传因素在肺癌的发生、发展、治疗反应、预后等中具有重要作用，因此开展肺癌的遗传研究对于肺癌易感人群的筛查、治疗选择、预后判断具有重要的意义，也成为肺癌基础研究领域中的一个重要内容。肺癌同多数肿瘤一样，不表现为典型的孟德尔式遗传，而是表现为微效基因的累加效应，即多基因遗传模式。单核苷酸多态性（single neucleotide polymorphism, SNP）是第三代遗传标志，已经被广泛地应用于肺癌的数量性状位点（quantitative trait loci, QTL）定位，并发现了大量相关多态性位点。现将近年关于SNP及其与肺癌遗传关联研究的进展作一简要综述。

## 1 SNP与肺癌关联研究的概述

SNP是指在染色体序列上由于单个核苷酸位置上存在转换、颠换、插入、缺失等变异而引起的DNA序列多态性，且至少在一个人群中这种变异的发生频率在1%以上^[4]^。SNP在基因序列中的高密度、代表性、遗传稳定性以及检测的易实现等特点体现了新一代遗传标记的优越性，使其被迅速接受从而成为遗传研究领域中极为活跃的内容。应用于肺癌的关联研究是该领域最活跃的内容之一。大量的SNP信息的发现催生了一些相关的数据库，如dbSNP、HGVbase、HapMap、EGPsnp、JSNP、HOWDY、GeneSNPs等，其中国际人类基因组单体型图计划（international HapMap project, www.hapmap.org）所建立的HapMap数据库能提供包括中国汉族人群在内的世界三大族群经过验证后的基因组多态性信息，与dbSNP成为最重要的参考数据库，与快速进展的检测技术一同推动着肺癌关联研究的进展^[5]^。

### 1.1 基于单个位点的关联研究

在已开展的SNP与肺癌的关联研究中，最初采用的策略是选择位于待选基因启动子区域或者编码子上可以导致编码氨基酸改变的多态性位点，也就是功能性SNP。通过这一策略，大量研究发现了一些与常见疾病具有关联的功能性SNP。但这一研究策略的一个重大缺陷是具有关联的单个位点的相对危险度一般只有1倍-2倍，在实际应用中的意义是非常有限的。但是这一阶段研究的一个更重要的意义是支持了肺癌为微效基因累加的结果这一假设，而且为后续的研究奠定了基础。

### 1.2 基于单体型水平的研究

随着对SNP研究的深入，发现了越来越多SNP位点间连锁不平衡的信息，HapMap计划的进展更是极大地推动着基因单体型信息的研究，从而为基于单体型水平的肺癌关联研究提供了可能。由于单体型更能反映疾病遗传的本质，而且技术成本低，对基因的覆盖率高，使其越来越为研究者所运用，并具有取代单个位点研究的趋势。近年开展的一些肺癌遗传相关研究，明显地体现了这种研究策略的优势^[6-9]^。

### 1.3 基于基因组水平的研究

基于单个基因水平的研究在实际应用中的意义是有限的，即使有研究者尝试应用大样本量的人群进行了多个基因的联合分析，结论仍然难以满足实际应用的需要^[10-12]^。针对肺癌系微效基因累加的假设，从基因组水平上开展基因与肺癌关联研究是当前迫切的需要，当前已经有研究者尝试从基因组水平上进行肺癌的关联研究，比如采用高通量芯片技术扫描、测序技术、高效液相色谱等，但从严格意义上来说还不属于全基因组水平的研究^[13-17]^。可喜的是当前新的大规模测序的方法先后出现，从而为实现这一目标提供了现实的可能性^[18]^。

## 2 SNP与肺癌的遗传易感性

肺癌的流行病学研究已经表明肺癌是一种典型的环境相关疾病，吸烟、环境污染等是导致肺癌发生的主要原因，但是研究也发现在同样的致癌因素作用下，只有少部分个体罹患肺癌，这现象表明了基于个体差异的遗传易感性的存在对于肺癌的发生具有重要作用。肺癌的家族聚集现象也表明了遗传因素对于肺癌发生的作用^[19]^。因此，开展肺癌的易感性研究对于肺癌易感人群的筛查具有重要意义，也成为肺癌遗传研究中得到最广泛研究的内容，涉及代谢酶基因、癌基因、抑癌基因、修复酶基因、凋亡基因等等。

### 2.1 代谢酶基因

肺癌的发生发展是一个多因素介入的多步骤过程，烟草及环境化学致癌物要在体内经过复杂的生物学代谢，因此肺癌易感性的不同在一定程度上可能取决于外来化学致癌物在体内的代谢差异。特别是在癌变的始动阶段，生物转化酶类的基因多态性对环境致癌物的致癌效应可能起着关键作用。体外实验发现谷胱甘肽-S-转移酶P1（glutathione-S-transferase P1, GSTP1）、髓过氧化物酶（myeloperoxidase, MPO）、细胞色素P450（cytochrome P450, CYP450）酶系等基因的多态性影响了烟草致癌物的代谢，也明显支持这一假设，因此目前生物转化酶的基因多态性是肿瘤易感性研究的热点之一^[20-24]^。目前开展的肿瘤代谢酶基因的研究主要集中在CYP450、谷胱甘肽转硫（glutathione-S-transferase, GST）酶系。

CYP450同功酶是一类含血红素的单加氧酶，也是体内最重要的Ⅰ相代谢酶，广泛存在于人体肺、肝、肾、脑等组织中。CYP450的作用的底物非常广泛，除了参与对烟草等前致癌物的代谢，而且还通过参与绝大多数内源性底物（如类固醇激素、维生素类、花生四烯酸、前列腺素、白介素等）的催化反应进而影响细胞的分化功能及细胞间信息传递。因此，CYP450酶系被认为是在肺癌形成中具有重要作用的一类代谢酶，关于其常见多态性与肺癌易感性关系的研究得到了广泛的研究，其中得到充分研究并认为与肺癌易感性相关的CYP450酶系有CYP1A1、CYP2A6、CYP2D6、CYP2A13、CYP2E1等。其中CYP2A13被认为是人呼吸系统中最常见的CYP450酶，而且多名研究者的研究结果高度一致，因此非常值得关注，其有可能在肺癌致癌物生成中具有重要意义。Wang^[25]^首次研究了CYP2A13常见多态性位点rs8192789（Arg257Cys）与中国汉族人群的肺癌易感性，研究发现该位点与肺腺癌具有相关性，CT+TT基因型较CC基因型降低了腺癌的易感性。之后法国的研究者^[26]^研究了高加索人群另一个多态性位点（Arg101Stop）与肺癌易感性的关系，研究发现该位点与小细胞肺癌具有相关性。Agostino研究发现多态性位点CT或TT基因型（Arg257Cys）的CYP2A13明显降低了对烟草前致癌物的代谢，与Wang的研究结果^[27]^是一致的。

GST是参与机体解毒的一组具有多种生理功能的蛋白质，催化机体内有害极性化合物与谷胱甘肽结合，也可由非酶结合方式将体内各种潜在毒性化学药物、致癌剂及亲脂性化合物从体内排出，达到解毒目的，同时还具有过氧化物酶和异物酶活性，结合胆红素和某些激素功能，从而参与机体内抗氧化和保护细胞内物质合成、储存和转运的过程。可见GST同工酶的表达和活性，在机体对致癌物等有害物质去毒、避免对遗传物质在进一步损伤的过程中有重要作用。当前得到广泛研究的GST代谢酶是GSTT1、GSTM1、GSTP1^[28-32]^。

除了CYP450酶系、GST酶系以外，得到广泛研究并认为与肺癌易感性具有相关性还有N-乙酰转移酶（N-acetyltransferase, NAT）^[33, 34]^、微粒体换氧化物酶（microsomal epoxide hydrolase 1, EPHX1）^[35]^。除了从致癌物生成角度研究肿瘤的易感性外，Chen^[36]^最近还发现了位于芳香烃受体基因（aryl hydrocarbon receptor, AHR）上的一个单倍体能够明显增加中国汉族人群对肺癌的易感性。具有高水平证据的几项多中心大样本研究都证明了位于人类15号染色体上的编码烟碱乙酰胆碱受体多个亚基的区域内特定SNP基因型与吸烟量、尼古丁依赖或肺癌发病风险相关，其中有两篇文章都发表在了Nature上^[37, 38]^。

### 2.2 癌基因

癌基因是指其编码的产物与细胞的肿瘤性转化有关的基因，其蛋白产物在信号转导、细胞生长、增殖、分化的调控方面起着重要作用，当这些调节或转导发生改变，即有可能影响人群对肿瘤易感性的不同。许多癌基因由于单碱基突变，导致癌基因产物中单个氨基酸的替换，而丧失其调节的活性。第一个被鉴定的人类癌基因是*ras*基因家族，其中*H*-*ras*、*K*-*ras*和*N*-*ras*是在人类肿瘤中最常见的癌基因，它们在大约15%的人类恶性肿瘤中被检出，包括50%的结肠癌和25%的肺癌。Kohno^[39]^研究了人*K*-*ras*基因常见多态性与肺癌易感性的关系，发现有两个常见多态性*K*-*ras1*、*K*-*ras6*与腺癌易感性具有一定相关性。Chin^[40]^研究发现位于*K*-*ras*基因3, 末端的一个SNP位点能够通过影响基因的表达进而影响到携带不同基因型的个体对肺癌易感性的不同。

### 2.3 抑癌基因

抑癌基因（tumor suppressor gene）是一类抑制肿瘤形成的负调节基因，其表达蛋白产物包括一系列调节因子，如转录调节因子、负调控转录因子、周期蛋白依赖性激酶抑制因子、信号通路的抑制因子、与发育和干细胞增殖相关的信号途径组分。因此，抑癌基因与调控生长的原癌基因协调表达在肿瘤的发生发展等方面起着重要作用。

*p53*基因是迄今发现与人类肿瘤相关性最高的抑癌基因，已知在几乎所有的肿瘤中p53都起着一定的作用，因此在肿瘤研究领域中得到了广泛研究，并且有望成为重要的肿瘤治疗基因。人类*p53*基因定位于17p13.1，其编码蛋白产物主要集中于核仁区，能与DNA特异结合，其活性受磷酸化调控。正常p53的一个重要生物功能是监视基因组的完整性。如有损伤，p53蛋白阻止DNA复制，以提供足够的时间使损伤DNA修复。如果修复失败，p53蛋白则引发细胞凋亡，现在已知p53在很多细胞凋亡信号通路中都扮演着十分重要的作用，包括膜凋亡信号、线粒体凋亡通路以及在细胞核内与凋亡相关的因子的转录与表达。Zhang等^[41]^研究了*p53*常见多态性位点rs1042522、Arg72Pro与肺癌易感性的关系，结果发现在高加索人群中携带Pro72Pro基因型的个体的肺癌的易感性增加。Mechanic^[42]^从单体型水平上研究了高加索人群和非洲人群中*p53*基因多态性与肺癌易感性的关系，他在对*p53*基因整体分析的基础上选择了16个常见SNP，包括标签SNP（tagger SNP）和功能性SNP，结果发现rs1042522、rs9895829、rs2909430、rs1625895、rs12951053在非洲人群中与肺癌易感性以及预后有关联，而由rs1042522、rs9895829、rs2909430、rs1625895组成的一个单体型OR值达到2倍以上。Li等^[43]^对公开发表的关于p53 Arg72Pro与肺癌易感性的关系的文献进行了荟萃分析，共纳入研究人群15, 857例，结果发现该位点与肺癌的易感性具有相关性，分层后发现这种相关性主要体现在亚洲及高加索人群、腺癌或者吸烟者中。

*p21*基因是另一个得到广泛重视的抑癌基因，Choi^[44]^分析了*p21*基因上两个常见SNP及其组成的单体型与肺癌易感性的关系，发现其与韩国人群易感性具有重要关联。其它得到研究并认为与肺癌有关联的抑癌基因还有p73^[45]^、Ras相关区域家族1基因（Ras-association domain family-1, *RASSF1*）^[46]^、过氧化物酶激活受体基因（peroxisome proliferator-activated receptor-*γ*, PPAR-*γ*）等^[47]^。

### 2.4 肿瘤侵袭、转移相关基因

肿瘤的侵袭与转移是其恶性标志和特征，也是影响治疗效果和导致患者死亡的主要原因。肿瘤侵袭、转移是一个极其复杂的多基因调控和多步骤发展过程，涉及到一系列相关基因的结构和功能的异常，肿瘤血管生成被认为是肿瘤侵袭和转移的一个重要机制。血管内皮生长因子（vascular endothelial growth factor, VEGF）及表皮生长因子受体（epidermal growth factor receptor, EGFR）在促进内皮细胞的分裂与增殖、促进血管生成中起着关键作用。Kim^[48]^研究了*VEGF*基因常见SNP在韩国肺癌人群中的分布，发现单个SNP与肺癌易感性没有相关性，但有一个单体型与肺癌易感性具有明显相关性。Zhai^[49]^研究了高加索人群VEGF基因多态性与非小细胞肺癌（non-small cell lung cancer, NSCLC）易感性的关系，纳入了1, 900例病例和1, 458例正常对照，也发现有一个常见单体型可能与高加索男性人群的腺癌有一定相关性。Jo等^[50]^研究了407例韩国肺癌人群和407例完全匹配的正常人群表皮生长因子受体2基因（epidermal growth factor receptor, *HER*-*2*）常见多态性与肺癌易感性的关系，结果发现3个常见SNP（-3444C＞T、- 1985G＞T、P1170A C＞G）与非吸烟、女性、非饮酒肺癌患者的易感性具有一定相关性。

基质金属蛋白酶（matrix metalloproteinases, MMPs）能降解细胞外基质（extracellular matrix, ECM）中的各种蛋白成分，因此有可能在肿瘤侵袭与转移中具有重要作用，Su^[51]^研究了高加索人群MMP多态性与肺癌之间的关系，研究纳入了2, 013例病例和1, 323例正常对照人群，发现4个具有连锁关系的常见SNP（rs1799750、rs3025058、rs2276109、rs652438）与肺癌发生具有相关性，单体型分析OR值达到3.65。Sauter的研究^[52]^也发现了在高加索人群中MMP1的常见多态性与肺癌的发生具有相关性。

### 2.5 凋亡基因

细胞凋亡（apoptosis）亦称程序化死亡（programmed cell death）或凋亡性细胞死亡（apoptosis cell death），是器官组织中细胞的一种正常的生理机制，是多细胞有机体调控机体发育、维护内环境稳定的细胞主动死亡过程。一般认为，肿瘤细胞的凋亡机制异常导致的细胞增殖和凋亡的失衡是导致肿瘤恶性生物学行为的一个基本机制，也被认为是影响肿瘤患者预后的一个重要的因素。所以可以推论凋亡相关基因的多态性有可能影响肿瘤的发生、侵袭与转移等。半胱天冬氨酸酶（caspase）是一组参与细胞凋亡的重要蛋白，Lee等^[53]^研究了韩国肺癌人群caspase7常见SNPs的分布，发现rs2227310位点及一个单体型（rs12415607、rs11593766、rs2227310、rs10787498）与肺癌发生具有相关性。来源于同样人群的研究^[54-56]^发现caspase-3、caspase-8、caspase-9常见多态性与肺癌均具有一定的相关性。

生存素基因（survivin）是新近发现的一个凋亡抑制蛋白家族新成员，它通过直接抑制caspase-3和caspase-7的活性和间接抑制caspase-9的活性阻断细胞凋亡，是具有抑制细胞凋亡和调节细胞分裂的双功能蛋白^[57, 58]^。现在已知survivin选择性表达于喉癌、肝癌、乳腺癌、卵巢癌等多种常见的恶性肿瘤，而在正常成人分化成熟的组织中基本不表达。因此在理论上survivin可以作为一个待选基因分析其常见多态性与肿瘤易感性的关系。Jang等^[59]^研究了韩国肺癌人群survivin常见多态性与肺癌发生的关系，发现在其选择的8个多态性位点中位于启动子区域的位点rs17884799及包括该位点的一个单体型与肺癌具有关联，因此可以推论位于该基因上游启动子区域的一个常见多态性位点可能影响了该基因的转录表达，进而影响了对肺癌的易感性。

### 2.6 修复酶基因

DNA修复是一系列与恢复正常DNA序列结构和维持遗传信息相对稳定有关的细胞反应，目前已知大概有超过100个修复酶参与DNA的修复作用，根据其修复机制的不同大体上可以分为5类，即直接修复、碱基切除修复、核苷酸切除修复、错配修复、双链断裂修复。因此，可以推论DNA修复酶的多态性有可能引起的DNA修复能力的差异，进而在一定程度上影响着肺癌遗传易感性的不同。目前关于修复基因多态性与肺癌易感性关系的研究是肿瘤易感性研究的一个热点，其中得到广泛研究并认为与肿瘤易感性具有相关性的有错配切除修复基因1（excision repair cross-complementing rodent repair deficiency complementation group 1, *ERCC1*）、错配切除修复基因2（excision repair cross-complementing rodent repair deficiency complementation group 2, *ERCC2*）、错配切除修复基因6（excision repair cross-complementing rodent repair deficiency complementation group 6, *ERCC6*）、X射线交叉补体1基因（X-ray cross-complementing group1, XRCC1）、X射线交叉补体4基因（X-ray cross-complementing group 4, *XRCC4*）、O_6_-甲基鸟嘌呤-DNA甲基转移酶（O_6_- methylguanine-DNA methyltransferase, *MGMT*）等^[15, 60-67]^。最近以中国汉族人群为研究对象的一项大样本量研究对比了*ERCC1*常见多态性分布在肺癌人群和正常人群的差异，共纳入了1 010例肺癌人群和1 010例正常人群，结果发现在检测的7个常见位点中，有两个位点（rs3212948、rs1007616）以及包括该位点的一个常见单体型与肺癌具有明确的相关性^[63]^，该项研究也是少有的针对中国人群的具有较高证据水平的研究。

除了上述得到广泛研究的修复酶基因，最近还发现了许多与肺癌具有相关性的其它修复酶基因，如8-羟基鸟嘌呤-DNA糖苷酶（8-oxoguanine DNA N-glycosylase 1, hOGG1）^[68-70]^、DNA依赖蛋白激酶（DNA dependent protein kinase, DNA-PK）、DNA连接酶（DNA ligase 1, LIG1）等^[71, 72]^。

### 2.7 其它与肺癌易感性相关的基因

肺癌的发生、发展是一个多原因多步骤的过程，除了广泛开展的关于代谢酶基因、抑癌基因、修复酶基因、转移相关基因、凋亡基因外，还有其它大量相关基因被发现与肺癌的发生有相关性。共济失调毛细血管扩张突变基因（ataxia telangiectasia mutated gene, *ATM*）基因在DNA的信号识别、修复中具有重要作用，针对中国汉族人群的一项应用较大样本量的研究发现该基因的常见多态性与非吸烟人群的肺癌易感性具有相关性^[73]^。其它被发现的与肺癌易感性相关的基因还有白介素1A基因（interleukin 1A, *IL1A*）、白介素1B基因（interleukin 1B, *IL1B*）^[74]^、多药耐药基因1（multidrug resistance 1, *MDR1*）^[75]^、丝氨酸/苏氨酸激酶15基因（serine/threonine kinase 15, *STK15*）^[76]^、β2肾上腺素受体基因（beta-2 adrenergic receptor, ADRB2）^[77]^、转录生长因子-β基因（transforming growth factor-β, *TGF*-*β*）^[78]^、DNA甲基转移酶-3B基因（DNA-methyltransferase-B, *DNMT3B*）^[79]^、5, 10-亚甲基四氢叶酸还原酶基因（5, 10-methylenetetrahyd rofolate reductase, *MTHFR*）^[80-82]^、血管紧张素转化酶基因（angiotensin-converting enzyme, *ACE*）^[83]^、白酪氨酸磷酸酶激酶基因（protein tyrosine phosphatase, *PTP*）^[84]^、脱氧嘧啶核酸内切酶基因（apyrimidinic endonuclease, *APE1*）^[85, 86]^、人硫转移酶基因1A1（human sulfotransferase 1A1, *SULT1A1*）^[87]^、甲基化鸟苷结合蛋白4（methyl-CpG binding domain protein 4, MBD4）^[88]^等。总之，关于SNP与肺癌易感性的研究是肺癌遗传研究领域中的一个热点，大量的研究发现了可能与肺癌发生具有关联的易感性SNP，这些有关联的SNP理论上有望成为筛选肺癌易感人群的分子标志物。需要指出的是，国内针对中国汉族人群也开展了广泛的研究，但是大多数研究采用的样本量过小，从而限制了研究结果的有效性，开展多中心的合作是解决这一问题的关键和必然途径。

## 3 SNP与肺癌的治疗及预后

不同肺癌患者间对药物治疗往往具有不同的反应，包括治疗效果及药物的毒性作用，这反映了个体间基因的差异会导致药理作用的不同，研究这种不同个体间基因的差异对于肺癌的临床个体化治疗具有重要意义^[89]^。铂类是肺癌的化疗中最常应用的药物，临床应用中不同个体间用药效果和毒性作用往往具有很大差异。Wu^[9]^研究了中国人群着色性干皮病D型基因（xerodemar pigmentosum group D, *XPD*）的常见多态性与进展期NSCLC患者应用顺铂治疗的3级-4级血液学毒性的关系，结果发现，位于156密码子上的多态性位点（rs238406）的常见基因型Arg156Arg能够明显地增加患者3级-4级血液学毒性，OR值达到3.24，而且在白细胞减少上作用更突出。Sun等^[90]^研究了*XRCC*及*XPD*常见多态性与进展期肺癌应用含铂类药物化疗的反应，结果发现位于XRCC 194位氨基酸密码子上的一个多态性位点与治疗反应具有明显相关性，但是该研究纳入样本量过小，限制了研究的证据学水平。Sohn^[91]^研究了应用EP方案化疗的小细胞肺癌患者多药耐药基因*MDR1*常见多态性与化疗反应之间的关系，结果发现位于外显子26上第3, 435核苷酸位点处携带CC基因型的患者较携带CT/TT基因型的患者具有更明显的化疗反应，由该位点以及第2, 677核苷酸多态性位点组成的单体型与化疗反应具有更明显的相关性。还有研究者^[92]^探讨了阴离子转运多态基因（anion-transporting polypeptides, *OATP1B1*）的常见SNP与NSCLC患者应用顺铂治疗的化疗敏感性及毒性作用的关系，也得出了有意义的结论。

SNP影响到肺癌患者对治疗的反应，必然会影响肺癌患者的生存期。吉非替尼是针对EGFR的特异性酪氨酸酶抑制剂，被广泛应用于进展期NSCLC的临床治疗，Liu^[93]^研究了*EGFR*基因常见多态性-216G/T、-191C/A、Arg497Lys与进展期NSCLC患者应用吉非替尼治疗效果的关系，发现-216位点携带T等位基因的患者获得了较长时间的无进展生存。Ma等^[94]^应用标签SNPs的方法研究了84例应用吉非替尼的中国汉族肺癌患者*EGFR*常见多态性与肺癌治疗结局的关系，研究发现rs2293347与患者的生存期具有明显相关性，可以成为一个预测指标。Heist^[95]^研究了维生素D受体基因（vitamin D receptor, *VDR*）与进展期NSCLC生存期之间的关系，共纳入294例病例，结果发现rs10735810位点CC基因型较CT/TT基因型患者具有更长的生存期，平均生存时间为21.4个月，进一步的单体型分析也得到同样的结果。Kim^[96]^研究了乳腺癌易感性基因1（breast cancer susceptibility gene 1, *BRCA1*）常见多态性与NSCLC生存期之间的关系，纳入了300例进行铂类为基础化疗的患者，结果发现了一个单体型与肺癌的生存期具有明显的相关性，作者认为该单体型可以成为判断患者化疗后生存期预测的生物标记物。Carcereny等^[97]^在一项纳入Ⅲ期临床试验的肺癌患者中，研究了芳香烃受体基因亚单位常见多态性与应用吉西他滨、铂类等药物的治疗反应及预后之间的关系，结果发现一个常见多态性位点rs1051730能够明显提高患者对化疗的治疗反应，并且延长无进展生存期及总生存期。其它研究还发现与肺癌患者生存期具有相关性的基因还有甘露糖结合凝集素基因（mannose-binding lectin 2, *MBL2*）^[98]^、MDR1^[99-101]^等。但是上述开展的几项研究由于样本量的原因导致证据水平不足。

## 4 基因组水平的研究

肺癌是SNP与疾病相关性研究领域中得到最充分研究的疾病，经过10余年世界范围内的研究工作，大量的侯选基因得到研究，研究人群覆盖几乎所有种族，发现了许多与肿瘤易感性、生存期、治疗反应等具有相关性的多态性位点，但一个共同缺陷是几乎所有有意义的SNP位点与疾病的关联度均是非常微弱的，虽然应用单体型分析方法进一步提高了研究效率，但单个基因的研究结果仍难以具有实际应用的意义^[102]^。目前已经有作者应用测序及芯片技术等方法从基因组水平上研究了肺癌的易感性，但从技术方法上仍然不属于全基因组水平上的肺癌群体遗传研究，而且样本量是有限的^[103-105]^。随着人类基因组计划的完成以及新一代基因分型技术的发展，使得从全基因组水平进行多态性与肿瘤关联研究具有现实的可能性^[106, 107]^。开展多中心合作、采用大样本人群、应用新一代大规模测序技术开展肺癌的群体遗传研究将是未来的研究方向。

## 5 问题与展望

关于SNP与肺癌关系的研究内容非常广泛，通过我们所作的简要回顾，已经可以明确地看到，作为新一代遗传标记，有着丰富数量的SNP能够解释肺癌的复杂遗传现象。肺癌作为一种典型的多基因遗传疾病，其在发生、发展、治疗反应、预后等方面所表现出来的遗传性差异就是由基因组上一系列相关基因的SNP群的共同作用导致的，是许多微效基因累加的结果。随着新一代大规模测序技术的进展，使得我们有望从全基因组水平对人类疾病进行群体遗传学分析，将有可能在基因水平上帮助我们揭示肺癌遗传现象的本质。
